# Exploring the challenges of social participation during COVID-19 in Saudi Arabia through an occupational therapy lens

**DOI:** 10.3389/fpubh.2024.1417857

**Published:** 2024-08-16

**Authors:** Areej Meny

**Affiliations:** ^1^King Saud Bin Abdulaziz University for Health Sciences, College of Applied Medical Sciences, Occupational Therapy Program, Jeddah, Saudi Arabia; ^2^King Abdullah International Medical Research Center, Western Region, Jeddah, Saudi Arabia

**Keywords:** social participation, COVID-19, participation in activities of daily living, social isolation, Saudi Arabia

## Abstract

**Aim:**

To measure the social participation of people in Saudi Arabia during the COVID-19 pandemic.

**Subject and methods:**

A cross-sectional survey was conducted among people in Saudi Arabia to measure their participation in social activities during the COVID-19 pandemic. A validated questionnaire of Social Participation Scale was used to collect data from five main regions in Saudi Arabia through social media platforms. Means, frequencies, and percentages were calculated through descriptive analysis. Mean scores and standard deviation of social participation of participants were also presented.

**Results:**

The total number of participants was 1,560 including Saudi (87.3%) and non-Saudi (12.7%) nationals. Most participants (60.2%) were female. The age of participants ranged between 16 and 24 years old. Around 62.1% of participants were married, 63.2% were educated, 48.4 were employed and 82% were from the Eastern region. Around 72% of participants earned <10,000 riyals per month compared to those (27.3%) who earned <5,000 riyals per month. A total 72.7% of the participants have been diagnosed by COVID-19. The mean score of social participation was 47.81 (SD = 0.27). Most participants (62.7%) reported that social participation was severely restricted. Around 68.2% of the participants were quarantined at the facilities.

**Conclusion:**

The social participation of people in Saudi Arabia had been severely restricted during the COVID-19 pandemic. An early assessment of people’s social participation would help to identify their problems and needs, to help them improve their participation in social activities and thus increase their overall quality of life.

## 1 Introduction

Social participation is an important aspect of a person’s life. It can be defined as the participation of individuals in meaningful activities where they interact with others in the society or community (1). Social activities may include visiting family, going out with friends, volunteering in the community, and attending weddings and social events (1).

Participation in social activities has positive impacts not only on mental health, but also enhances activities of daily living among adults (2). It is reported that psychological wellbeing has been increased among people who actively participated in social activities (3). A strong association was also found between social participation and health outcomes (4–6) leading to increased social wellbeing (7). Azizi (8) found that there was a strong relationship between happiness and participation in different types of social events. Although social participation affects individuals’ lives positively, it had been reduced during the pandemic of COVID-19.

The COVID-19 pandemic started in Saudi Arabia in 2019. The first case was confirmed in March 2020. It has been reported that the number of confirmed cases and deaths have increased to more than 262,000 and 2,772 (9), respectively. One of the positives developments was made by the Ministry of Health was releasing a daily report about the affected cases, number of death and number of cases recovered and taking immediate actions accordingly (10). Due to the rapid spread of the pandemic, the Saudi government established some preventive policies to reduce the rate of infection. For example, traveling internally and externally was restricted. Schools, universities, malls, and mosques were closed and lockdowns, curfews, social events were canceled, religious activities like Hajj and Umrah were suspended and social/ physical distances were implemented (11).

In the mid of December in 2020, Pfizer-BioNtech vaccine was approved to be used in Saudi Arabia and vaccination processes were started in the same month. In February 2021, Oxford-AstraZeneca vaccine was also approved, and the government decided to vaccinate 10 million individuals, including citizens, residents, and refugees between February and September 2021 (12). The government prioritize delivering vaccination to population starting by healthcare providers, followed by older adult people above 65, obese people, patients with immunological conditions and patients with chronic diseases (12). In June 2020, few people who were vaccinated were permitted to perform Hajj with restrict procedures whereas in August 2021 many vaccinated people were allowed to practice Umrah under specific preventive measures (10). People were also allowed to visit Saudi Arabia under the condition of having the vaccine and had negative result of COVID-19. From February 2022, it was mandatory to provide vaccine certificate with the third does to enter and exit from Saudi Arabia and all travelling restrictions related to COVID-19 were released by February 2022 (10).

The Ministry of Health also took a proactive step by establishing walk-in respiratory clinics like Tatamman for serving people who experienced COVID-19 symptoms. These clinics were equipped with high qualified health care professionals and supplied with the required equipment for diagnosis (10). The ministry also developed several applications like Tawakalana to identify the positive cases and vaccination status before entering to public and governmental organizations (12).

Limited participation in the aforementioned activities has been proven to not only affect mental health but also decrease psychological wellbeing (13, 14) and participation in other occupations such as self-care and leisure (5, 15, 16). Participants from 74 countries reported that they were unable to participate in leisure activities such as outdoor activities and sports (15).

Several studies assessed social participation and how it has been affected during COVID-19 pandemic globally (4, 13, 17–19). Ammar et al. (4) revealed that there was a significant reduction in social participation with family, friends, and other leisure activities among people in Asia, Africa, Europe, and other countries during COVID-19 pandemic due to enforced home confinement. The majority of them reported lower life satisfaction due to less participation in social activities (4, 17). Additionally, it is reported that there was an association between social isolation and poor life satisfaction with some level of stress among adults aged between 18 and 84 years old (13). Similarly, a recent study from Israel discussed that secondary complications would develop among people in Israel because of limited participation in social activities, a significant decrease in quality of life, and a high level of psychological distress (17). Depression, anxiety, disturbance in sleep, and reduction in physical activities during COVID-19 were also narrated in a recent systematic review study (19).

Despite several publications, there was little literature found about the effect of COVID-19 on people’s social participation in Saudi Arabia. Therefore, this study aims to assess the impact of COVID-19 on the social participation of people in Saudi Arabia. The outcomes of this study would help identify the level of social participation during COVID-19 to establish some strategies for early intervention and its implementation, which may assist in reducing further complications of social isolation.

## 2 Materials and methods

Ethical approval (PNU-DRI-Targeted-20-011) was obtained from the relevant authorities. Data was collected online using convenience sampling techniques. SurveyMonkey was used as a tool for data collection. After designing the questions on SurveyMonkey the electronic version of the questionnaire was distributed through online platforms, including WhatsApp, Facebook, LinkedIn, and Twitter. Since the literacy rates in Saudi Arabia was 98% in 2020 (20) so the use of social media in the general population is well-versed. Participant information statement with the purpose of the study was added in the questionnaire. Consent was obtained from all participants before starting to fill out the questionnaire. The duration of data collection was from March 2020 to January 2021.

### 2.1 Participants

Inclusion criteria for participants were that they had to fluently speak and understand Arabic and the English Language. They were aged between 16 and 65 years old. Confidentiality of participants was maintained.

### 2.2 Tools

Data were collected using two forms of questionnaires.

#### 2.2.1 Demographic data collecting form

Sociodemographic information like age, gender, education, employment status living area etc. were included in this part.

#### 2.2.2 Social participation scale: English and Arabic version

Social Participation Scale was developed (21) to measure SP in rehabilitation, stigma reduction, and social integration programs. Arabic and English versions were used for data collection. It is an interview-based instrument, including closed structured questions.

The total score of the scale is 90 and the minimum is 0. The classification of SP restrictions is as follows. Participants who score from 0 to 12 would have no significant restrictions, 13–22 mild restrictions, 23–32 moderate restrictions, 33–52 severe restrictions, and 53–90 extreme restrictions.

### 2.3 Data analysis

Data was entered and analyzed using SPSS version 21.0. Means, frequencies, and percentages were calculated through descriptive analysis. Mean scores and standard deviation of SP of participants were also presented.

## 3 Results

The total number of participants was 1,560 and the majority of them (60.2%) were female. Around 53.6% of participants were aged between 16 and 24 years old. A large number of participants (87.3%) were from Saudi nationals compared to 12.7% of non-Saudi nationals. Most of the participants (62.1%) were married whereas only 3% were divorced or separated. A total 82% of the participants were from Eastern (40.1%) and Western (42.2%) regions followed by those from Central, Northern, and Southern ones. The majority of the participants were educated (63.2%) holding bachelor’s degrees and above. Nearly half of the population was employed (48.4%). Almost 72.7% of the participants were affected by COVID-19. Most of the participants (72.7%) reported their income to be under 10,000 riyals per month compared to those (27.3%) who reported earning <5,000 Saudi riyals per month (see Table 1).

**Table 1:** Demographic data of participants (*n* = 1,560).

Variables	Frequency	Percent
Age		
16–24	836	53.6
25–34	378	24.2
35–44	220	14.1
45–54	97	6.2
55–64	29	1.9
Gender		
Male	621	39.8
Female	939	60.2
Marital status		
Married	968	62.1
Single	545	34.9
Divorced	36	2.3
Separated	11	0.7
Education status		
Illiterate	8	0.5
Primary	18	1.2
Middle	100	6.4
High school	448	28.7
College and above	986	63.2
Nationality		
Saudi	1,362	87.3
Non-Saudi	198	12.7
Employed		
Yes	755	48.4
No	805	51.6
Affected by COVID-19		
Yes	1,134	72.7
No	426	27.3
Region		
Central	156	10.0
Eastern	625	40.1
Northern	95	6.1
Southern	19	1.2
Western	665	42.6
Income per month		
<5,000	1,134	72.7
5,000–10,000	426	27.3

Table 2 below shows that the mean scores of social participation of participants is 47.81 (SD = 0.27), which illustrates they have severe restrictions.

**Table 2:** Mean scores of social impact scale.

Social impact scale	*N*	Mean	Std. error of mean	Minimum	Maximum
	1,560	47.81	0.27	23.00	84.00

Table 3 indicates that the majority of participants (62.7%) reported that their social participation was severely restricted. More than 68% of the participants reported that they were quarantined at the facility compared to those (25.7%) who were quarantined at home.

**Table 3:** Categorized_social_impact and quarantine.

	Frequency	Percent	Valid percent	Cumulative percent
Categorized social impact				
Moderate restriction	85	5.4	5.4	5.4
Severe restriction	978	62.7	62.7	68.1
Extreme restriction	497	31.9	31.9	100.0
Quarantine				
Never	95	6.1	6.1	6.1
Home	401	25.7	25.7	31.8
Facility	1,064	68.2	68.2	100.0

## 4 Discussion

Social participation is one of the key factors that enhances people’s mental health and psychological wellbeing. However, it is understandable that it might be negatively affected during COVID-19 due to the implementation of governmental policies regarding lockdowns. Therefore, this study aims to measure the social participation of people in Saudi Arabia during COVID-19.

The findings of the present study showed that most participants reported severe social restrictions during COVID-19. The findings of this study support the findings of several other studies (4, 13, 22). For instance, one multicentre study found that there was a significant decrease in social participation and life satisfaction within the general population during the COVID-19 pandemic (4). Another research also reported an association between limited participation in social activities and a decrease in wellbeing and life satisfaction among participants during COVID-19 (13). Consequently, research by (19) found that people in Asia, Europe, and America who had limited social participation had negative impacts on their psychological wellbeing such as increased anxiety, depression, sleep disturbance, and reduced physical activities (19). A strong correlation between engaging in physical activities and mental wellbeing was also reported by the Italian population (22).

One of the reasons for the decrease in social participation during the pandemic could be due to lockdowns and curfews that were imposed during the background of the increasing number of affected cases (23). Another reason could be that due to being infected with the virus (24) people were indeed unable to participate in meaningful activities such as going to the gym/mall, and visiting family which had a negative impact in their quality of life, satisfaction and wellbeing (25, 26). Exploring the Saudi culture, it is known that people in Saudi Arabia value religious activities like praying, caring for families, and social gatherings, particularly during the religious festival of Eid. During COVID-19, people were unable to perform these activities due to the lockdown, which led to a loss of participation in significant social activities, and they had to adapt forcibly to the new situation by participating in the available activities at home (26). Such temporary disruption in everyday life may lead people to feel occupationally deprived of participation in different occupations due to the restricted participation in important meaningful activities, which was out of their control (27).

This study also revealed that a large number of participants (68.2%) were quarantined at a facility at some point in time during the COVID-19 pandemic. Due to the increasing number of COVID-19 cases (336,004 at the end of 2020) (9), the Saudi government implemented several strategies to control the pandemic, one of which was implementing quarantine to reduce spread. Alfaifi et al. (25) reported that healthcare providers and non-healthcare providers who were affected by COVID-19 were quarantined in facilities for 14 days. Although the implementation of quarantine helped to reduce infection (25), being quarantined for a long time would inevitably affect social participation. It was further reported that people who were not able to participate in different activities like gathering with families, and attending religious activities were at high risk of having not only psychological problems (14, 25, 28), but also physical, dietary, behavioral, and lifestyle issues (29–31). It is also reported that people from 74 countries experienced difficulties participating in outdoor activities (volunteering) and traveling (locally and internationally) compared to engaging in indoor activities (e.g., watching TV and painting) (15).

## 5 Conclusion, implications, and limitations

Social participation of people in Saudi Arabia was severely restricted during the COVID-19 pandemic based on the outcomes of this study. Early assessment of people under restricted social participation, can help in identifying their problems and needs. This can eventually assist them in improving their participation in social activities based on their wishes to enhance their general wellbeing.

Although this was a large-scale nationwide Saudi study, it had certain limitations. The research was based on quantitative data mainly, whereas including views and experiences of the affected population could have provided better insight. The study tapped into the general state of social participation of the population without digging deeper into the individual categories like productivity, self-care, and leisure due to the time. Association between variables like social participation and sociodemographic data were not explored due to the feasibility issues. Conducting further studies in the future, with improved designs like mixed methods, including the detailed assessment of sub-categories of social participation and including the participants’ feedback about ways to improve their social participation can provide better insight.

## Data availability statement

The raw data supporting the conclusions of this article will be made available by the authors, without undue reservation.

## Ethics statement

The studies involving humans were approved by the Scientific Research Ethics Committee at Prince Nourah bint Abdulrahman (PNU-DRI-Targeted-20-011). The studies were conducted in accordance with the local legislation and institutional requirements. The participants provided their written informed consent to participate in this study.

## Author contributions

AM: Conceptualization, Investigation, Methodology, Writing – original draft, Writing – review & editing.
